# An Analysis of Catastrophic Out-of-Pocket Health Expenditures in Ghana

**DOI:** 10.3389/frhs.2022.706216

**Published:** 2022-03-22

**Authors:** Fuseini Sataru, Kwame Twumasi-Ankrah, Anthony Seddoh

**Affiliations:** ^1^Center for Health and Social Services, Accra, Ghana; ^2^Department of General Studies, School of Human Development, Heritage Christian College, Accra, Ghana; ^3^World Bank, Accra, Ghana

**Keywords:** catastrophic health expenditure, out-of-pocket payments, financial risk protection, inequalities, Ghana

## Abstract

**Introduction:**

Ghana implemented a universal health coverage scheme aimed at attaining financial risk protection against catastrophic out-of-pocket health expenditures. The effort has yielded mixed benefits for the different socio-economic profiles of the population. The present study estimates the incidence of catastrophic payments among Ghanaian households.

**Methods:**

The study analyzed the round seven dataset of the Ghana Living Standards Survey collected between 2016 and 2017. We estimated the incidence and intensity of catastrophic payments for total household consumption and non-food consumption for a range of thresholds. The analysis further weighted the measures of catastrophic payments to determine the distribution sensitivity.

**Results:**

As the threshold increased from 10 to 25% of total household consumption, the incidence of catastrophic payments dropped from 1.0 to 0.1%. At the 40% threshold of non-food consumption, the estimated incidence was 0.2%. For both total household consumption and non-food consumption, the concentration indices were negative at all the thresholds. The results were indicative of a higher concentration of financial catastrophe among the poorest households and significant inequalities in the incidence between the poorest and richest households.

**Conclusion:**

The study confirmed the declining trend in the general incidence of catastrophic health expenditures in Ghana. However, the incidence and risk of financial catastrophe remained disproportionately higher among the poorest households, which is instructive of gaps in financial risk protection coverage. The Ghana National Health Insurance Scheme must therefore strengthen its targeting and enrolment of this sub-population group to reduce their vulnerability to catastrophic payments.

## Introduction

Globally, a billion people are projected to suffer from financial ruin due to out-of-pocket (OOP) payments for healthcare services at the time of need (1). This is indicative of the inability of national health financing systems, especially of low- and middle-income countries (LMICs), to provide adequate financial risk protection (FRP) against the costs of healthcare services (1–6). Thus, FRP, a fundamental dimension of the Sustainable Development Goal (SDG) on Universal Health Coverage (UHC) (i.e., SDG Target 3.8), has emerged as a global health policy priority and national health systems' goal (1, 7, 8). Lately, an aspired objective of health financing system reforms seek to advance the access of healthcare services based on need and contribution to the costs according to means in both developed and developing countries (1, 3–8). Defined as the ability to access healthcare services without the risk of financial hardship (1, 2, 9), FRP is achieved when there is total population-wide protection against catastrophic and impoverishing OOP health spending by households (1, 3–5, 9). The former is the focus of the present study. OOP payments, the upfront outlays on services used to obtain care for a household health need, determine the level of financial catastrophe. For poor and vulnerable households, such payments above 10 or 25% of their total expenditure or income or 40% of their non-food expenditure can result in catastrophic health expenditures (CHEs); that is SDG indicator 3.8.2 (1, 10, 11). However, not all OOP expenditures incurred by households lead to a financial catastrophe (1, 11, 12).

Trend-wise, while OOP health payments have been declining globally, as a proportion of household income, they have remained unchanged (8). Consequently, the incidence of catastrophic payments has increased over time (1, 8)—for instance, between 2000 and 2015, the global incidence increased, on average, annually by 3.6 and 5.3% at the 10 and 25% thresholds of total household consumption (1). Wagstaff et al. (8), in a cross-country comparative analysis, showed an increasing trend in the incidence of households' financial catastrophe in 48 of 94 study countries for both measures of total household consumption and non-food consumption. The study, notably, highlighted the parallels in the slow decline and rapid increases in incidence in different global regions. Although persistent in countries of all income levels, the socio-economic inequalities in financial catastrophe are disproportionately large in the LMICs of Africa and Asia where about 90% of the incidences occur (1, 7, 8). Njagi et al. (13), in a systematic review of CHEs in sub-Saharan Africa (SSA), found, on average, 23% incidence at the 10% threshold of household income and 17% when the 40% threshold of non-food expenditure is applied. The review revealed significant variations between the general population incidence and the elevated incidences among the populations with the need for specific diagnostic services, such as for HIV/ART, malaria, and TB. Across the region, household economic or income status, type of healthcare provider, type of illness, characteristics of household members, geographical location and distance to health facility, social insurance scheme membership, and household size and composition were the most consistent determinants of catastrophic health spending (13).

In Ghana, concerted efforts toward the achievement of the second objective of its National Health Policy—to address inequities in financial protection by ensuring sustainable financing for healthcare delivery and financial protection for the poor (14)—have produced mixed results—for instance, the incidence of CHEs declined from 15% in 1995 to 1.9% in 2015 at the 25% threshold of non-food consumption. At a projected 96.2% probability of achieving universal FRP coverage by 2030, Ghana is well ahead of most of its SSA peers in progress toward SDG indicator 3.8.2 (15). Two factors have been credited with this progress (16). Foremostly, the introduction of a national health insurance scheme (NHIS) in 2003 has since pursued a pro-poor prepayment policy by targeting the bottom two quintiles of the population and disadvantaged groups (17–19). The scheme has been extensively described in empirical literature (20–23). Also, improved living conditions were attributed to a generous decline in the national poverty prevalence (16). Despite this progress, in 2015, the poorest households still experienced 0.7 percentage points more in financial catastrophe compared with the richest (15). This is indicative of the persistence of socio-economic inequalities in the incidence (15, 16).

Intensified efforts toward UHC in LMICs have included a focus on building a coherent body of literature on the dimensions of FRP (24, 25). In Ghana, this can be traced to the early years following the introduction of the NHIS. Few empirical studies measured CHEs before this period. Of note is the study by Akazili et al. (16) on the baseline indicators of CHEs prior to the nationwide uptake of the scheme. Hence, the literature is predictably skewed toward evaluating the effects of health insurance on the level of catastrophic payments incurred by households. These studies, however, were limited in their application. They mainly assessed the incidence of financial catastrophe between insured and uninsured groups (17, 26–30) and for specific services or conditions (26, 29). Furthermore, the relevant methods employed were limited to one to two thresholds for the measure of mostly households' non-food expenditure (17, 26–30). To a degree, the exception is the trend analysis by Zhang et al. (15) using multiple datasets from the Ghana Living Standards Survey (GLSS). Even so, their estimation was at only the 25% threshold of households' non-food consumption (15). Saksena et al. (7) and Akazili et al. (16) underscored the importance of periodic monitoring of CHEs using nationally representative household surveys to assess the general situation, patterns, and trends over time in a country. The present study, to the best of our knowledge, is the first to apply a range of thresholds to estimate the general population incidence of catastrophic payments incurred by Ghanaian households for both total household consumption and non-food consumption using health insurance era data from the latest round of the GLSS.

## Methods

### Data

The data for this analysis is from the GLSS 7 which was conducted between 2016 and 2017. The GLSS is a multipurpose nationally representative survey that provides information on households' living conditions, income, and expenditure and general well-being. The survey used a two-stage stratified sampling design where the primary sampling units consisted of 1,000 enumeration areas. This produced a total survey sample size of 15,000 households, a 17% reduction from the GLSS 6. Overall, the survey response rate was 93.3%, representing 14,009 households of the sample size. A detailed description of the sampling design is available from the GLSS main report (31). The GLSS 7 collected information on the daily and occasional household expenditures. In the survey, consumption was measured using an extensive set of modules capturing home production of food as well as market purchases of goods and services and the use of valued consumer durables. The OOP spending data covers both direct and indirect expenditures on inpatient and outpatient services at public and private facilities. This, however, excluded third-party payments and insurance premiums and reimbursements. Outpatient services in the survey was defined as facility-based services provided to non-admitted patients by certified health facilities. Total expenditure on health, on the other hand, was defined as the sum of public and private health expenditures for the provision of both preventive and curative services.

### Data Preparation

To get nationally representative estimates, we used the GLSS 7 dataset weights in our analysis. In addition, we took the regional stratification into account and identified the locations, *i*.*e*., rural/urban drawn as a primary sampling unit within the strata (location). There are 14,009 households within the sample, where a household is defined as the number of persons living and eating meals together in the same dwelling (household size). Living standards was measured by total household consumption in thousands of Ghana cedis. OOP payments was measured by medical out-of-pocket payments, that is, the net of health insurance reimbursements during the past 12 months prior to the survey. Household OOP payments which include both formal and informal payments were included in the measure of households' consumption (32). We were guided by previous studies that used total household consumption as proxy for income on account of the high degree of unpredictability of households' incomes across time in low-income settings such as Ghana (8, 32, 33). Therefore, relative to income, the use of total household consumption significantly reduced the likelihood of variation and underestimation of the results (8). For non-food consumption, in relation to healthcare payments, financial catastrophe was defined as the proportion of expenditure remaining after outlays on food, also termed as “non-discretionary expenditure.” Households' spending after expenditures on food is commonly used as a living standards indicator in low-income settings (32). For both total household consumption and non-food consumption, we estimated the incidence and intensity for a range of thresholds, above which financial catastrophe can occur for households. The applied thresholds were 5, 10, 15, 25, 30, and 40% and the incidence of financial catastrophe was assessed at the household level. This is important for evidenced-informed policy and decision making (8, 32). However, we report the 10 and 25% thresholds for total household consumption recommended by the SDGs and the 40% threshold for the non-food consumption estimates commonly used in empirical studies (1, 8, 32). Stata (version 15) and The Automated DEC Poverty Tables were used to prepare and analyze the data.

### Measuring the Incidence and Intensity of Catastrophic Payments

We estimated the incidence of catastrophic payment headcount as the proportion of households' healthcare payments expressed as share of the total household consumption and non-food consumption above a range of thresholds. In decreasing order, the cumulative fraction of households is ordered by the ratio *T*/*x*. Let *T* be the per capita household out-of-pocket spending on healthcare. Let *x* be the per capita living standards proxy that is used in the standard assessment of poverty, that is, household consumption. For convenience, we refer to the living standards variable as household consumption. *H* is the households with healthcare budgets above the threshold *z. E* is an indicator which equals 1 if *Ti*/*xi* > *z* and 0 if otherwise. *N* equals the sample size (32, 34). The headcount was then computed as follows:


$$\begin{array}{l} H=\frac{1}{N}\overset{N}{\underset{i=1}{\sum }}E_{i} \end{array}$$

To estimate the intensity of financial catastrophe which is not captured by the headcount, the catastrophic payment overshoot was estimated. As a proportion of total expenditure, the overshoot shows the average degree by which households' healthcare payments exceeded threshold *z* (32, 34). The household overshoot was defined as follows:


$$\begin{array}{l} O_{i}=E_{i}\left[\left(\frac{T_{i}}{x_{i}}\right)-z\right] \end{array}$$

The overshoot then is the average:


$$\begin{array}{l} O=\;\frac{1}{N}\overset{N}{\underset{i=1}{\sum }}O_{i} \end{array}$$

We defined *O* = *H* × *MPO*: the catastrophic overshoot equals the fraction with catastrophic payments times the mean positive overshoot (MPO). The MPO measures the intensity of catastrophic payments, that is, the average excess of health payment budget share of those households with catastrophic payments (33, 35).

### Distribution-Sensitive Measures of Catastrophic Payments

To determine if the catastrophic headcount or gap is concentrated among the poor or rich, the analysis weighted the measures of catastrophic payments. This is necessitated by the insensitivity of the headcount and overshoot to the distribution of catastrophic payments. The concentration indices were estimated as *Ei* and *Oi*, where *C*_*E*_ is the concentration index of *Ei*, and *C*_*O*_ is the concentration index of *Oi*. The indices are negative when *C*_E_<0 and *C*_O_<0 and indicative of a higher concentration of catastrophic payments among the poorest households (32, 34). The headcount and overshoot were multiplied by the complement of the respective concentration indexes to adjust the distribution of the catastrophic payments (32, 35). The weighted headcount and overshoot were estimated as:


$$\begin{array}{l} H^{W}=H\;.\;\left(1-\;C_{E}\right) \end{array}$$

and


$$\begin{array}{l} O^{W}=O\;.\;\left(1-\;C_{O}\right) \end{array}$$

## Results

The summary statistics of health payments by household socio-demographic and socio-economic characteristics is presented in Table 1. There were three household characteristics: area of residence, region, and poverty status—for instance, with GHȼ 4,217.9, rural households spent more on healthcare than urban households. In terms of poverty status, the well-off households had a higher gross per capita consumption (GHȼ 4,376.1) than the poor households (GHȼ 1,067.1). By region, there were variations in total payments made by households, which was lowest in the Upper West region (8.6%) compared with Greater Accra (48.9%). In terms of expenditure on outpatient services, rural households, on average, spent more (9.4%) on healthcare payments than urban households (3.8%). The households spent a proportion of 6.6% of their finances on outpatient services. The total expenditure on health by households, on average, was 21.7%, amounting to an average per capita consumption (net) of GHȼ 3,134.7.

**Table 1:** Health payments by household socio-demographic and socio-economic characteristics.

	**Per capita consumption, gross (GHȼ)**	**Expenditure on outpatient services (%)**	**Total expenditure on health (%)**	**Total health payment (%)**	**Per capita consumption, net of payments (GHȼ)**
**Area of residence**					
Urban	2,077.5	3.8	16.0	19.8	2,057.7
Rural	4,217.9	9.4	27.2	36.6	4,181.3
**Region**					
Western	2,832.9	4.9	16.1	21.0	2,811.9
Central	3,157.7	8.5	26.7	35.2	3,122.5
Greater Accra	5,739.1	15.2	33.7	48.9	5,690.2
Volta	2,097.9	3.5	26.4	29.9	2,068.0
Eastern	3,037.3	5.1	24.1	29.2	3,008.0
Ashanti	3,592.4	7.0	20.3	27.3	3,565.2
Brong Ahafo	2,404.3	3.2	10.9	14.1	2,390.2
Northern	1,519.9	2.7	14.9	17.6	1,502.3
Upper East	1,426.5	3.5	17.7	21.3	1,405.2
Upper West	1,138.9	1.0	7.7	8.6	1,130.3
**Poverty status**					
Poor	1,067.1	2.5	10.8	13.2	1,053.9
Non-poor	4,376.1	9.1	28.0	37.0	4,339.1
Total	3,163.0	6.6	21.7	28.3	3,134.7

Table 2 presents the measures of the incidence and intensity of catastrophic payments for healthcare services in Ghana. As the threshold increased from 10 to 25% of the total household consumption, the estimate of the incidence of catastrophic payments (*H*) dropped from 1.0 to 0.1% and the mean overshoot from 0.1 to 0.0%. Standard errors were small relative to the point estimates, considering the large sample size of the GLSS 7. Unlike the headcount and the overshoot, the MPO among those households that exceeded the threshold need not reduce as the threshold increased. As shown in Table 3, when defined using non-food consumption, the estimate of the incidence of catastrophic payments (*H*) at the 40% threshold was 0.2%, and the mean overshoot was 0.0%. When defined using both total household consumption and non-food consumption at the 5, 10, and 15% thresholds, the catastrophic headcount for the lowest quintile was higher than for the highest quintile—for example, using total household consumption at the 5% threshold, the headcount for the lowest quintile is 6.0% compared with 3.1% for the highest quintile.

**Table 2:** Incidence and intensity of catastrophic health payments—total household consumption.

	**Threshold budget share**					
	**5%**	**10%**	**15%**	**25%**	**30%**	**40%**
**Headcount**						
Lowest quintile	6.0	1.8	0.6	0.2	0.1	0.1
2	2.8	1.0	0.4	0.1	0.0	0.0
3	2.3	0.3	0.1	0.1	0.0	0.0
4	2.9	0.8	0.4	0.1	0.1	0.0
Highest quintile	3.1	1.2	0.6	0.0	0.0	0.0
Average	3.4	1.0	0.4	0.1	0.0	0.0
**Overshoot**						
Lowest quintile	0.3	0.1	0.1	0.0	0.0	0.0
2	0.1	0.1	0.0	0.0	0.0	0.0
3	0.1	0.0	0.0	0.0	0.0	0.0
4	0.1	0.1	0.0	0.0	0.0	0.0
Highest quintile	0.2	0.1	0.0	0.0	0.0	0.0
Average	0.2	0.1	0.0	0.0	0.0	0.0
**Mean positive overshoot**						
Lowest quintile	4.6	6.2	8.6	12.2	15.0	13.6
2	5.3	6.9	8.0	12.3	19.7	20.8
4	4.7	8.1	9.2	11.6	11.9	19.3
Average	4.7	6.7	7.6	10.0	13.9	16.6

**Table 3:** Incidence and intensity of catastrophic health payments—non-food consumption.

	**Threshold budget share**					
	**5%**	**10%**	**15%**	**25%**	**30%**	**40%**
**Headcount**						
Lowest quintile	15.0	7.6	4.3	1.6	1.2	0.6
2	9.8	4.4	2.3	1.0	0.5	0.2
3	9.0	3.3	1.2	0.3	0.2	0.1
4	8.7	3.1	1.5	0.5	0.4	0.2
Highest quintile	7.0	3.1	1.8	0.6	0.3	0.0
Average	9.9	4.3	2.2	0.8	0.5	0.2
**Overshoot**						
Lowest quintile	1.3	0.8	0.5	0.2	0.2	0.1
2	0.7	0.4	0.3	0.1	0.1	0.0
3	0.5	0.2	0.1	0.0	0.0	0.0
4	0.5	0.3	0.2	0.1	0.1	0.0
Highest quintile	0.5	0.3	0.2	0.0	0.0	0.0
Average	0.7	0.4	0.2	0.1	0.1	0.0
**Mean positive overshoot**						
Lowest quintile	8.9	10.5	11.9	14.5	13.7	14.4
2	7.5	9.4	10.7	9.8	11.6	12.3
3	5.4	6.7	10.0	13.9	16.1	13.3
4	6.0	8.5	10.6	13.9	14.3	10.1
Highest quintile	7.2	8.6	8.3	6.9	7.6	13.4
Average	7.2	9.1	10.7	12.2	12.9	13.1

Table 4 shows the concentration indices, rank-weighted headcount, and overshoot measures. The distribution of catastrophic payments depends on whether health payments are expressed as a share of total household consumption or of non-food consumption. Using total household consumption, the concentration indices increased from −0.141 at the 5% threshold to −0.040 at the 15% level. This then decreased from −0.223 at the 25% threshold to −0.567 at the 40% level. When assessed using non-food consumption, the concentration indices decreased from −0.201 at the 10% threshold to −0.391 at the 40% level. The negative concentration indices for both total household consumption and non-food consumption at all the thresholds were indicative of a higher concentration of catastrophic payments among the poorest households. The results were also suggestive of significant inequalities in catastrophic payments between the poorest and richest households. This is evidenced in the increased concentration indices with the threshold for the higher thresholds (25, 30, and 40%).

**Table 4:** Distribution-sensitive catastrophic payment measures.

	**Threshold budget share**					
	**5%**	**10%**	**15%**	**25%**	**30%**	**40%**
**Total household consumption**						
Concentration index	−0.141	−0.114	−0.040	−0.223	−0.344	−0.567
Rank-weighted headcount	3.890	1.110	0.444	0.127	0.066	0.029
Concentration index	−0.134	−0.125	−0.172	−0.361	−0.407	−0.493
Rank-weighted overshoot	0.181	0.075	0.038	0.014	0.010	0.005
**Non-food consumption**						
Concentration index	−0.145	−0.201	−0.223	−0.256	−0.339	−0.391
Rank-weighted headcount	11.339	5.159	2.731	1.007	0.678	0.338
Concentration index	−0.225	−0.265	−0.292	−0.359	−0.395	−0.446
Rank-weighted overshoot	0.877	0.495	0.309	0.133	0.091	0.046

## Discussion

This study sets out to assess the incidence of catastrophic health payments among Ghanaian households. Overall, as share of both total household consumption and non-food consumption, our results are marginally lower than that reported by other studies that also analyzed national household survey datasets. Most importantly, they corroborate a trend. Akazili et al. (16), in their baseline indicator assessment using the user fee era dataset from round 5 of the GLSS (2005/2006), reported incidences of 5.16 and 2.56% at the 10 and 20% thresholds of total household expenditure. Wagstaff et al. (8), in a retrospective observational cross-country assessment, reported 3–5 and 0.2–0.6% incidence at the 10 and 25% thresholds of total household consumption for Ghana using datasets from 1991 to 2005. Zhang et al. (15) analyzed datasets from four rounds of the GLSS covering both the user fees and health insurance eras and found that, at the 25% threshold of household non-food consumption, the incidence was 1.9% in 2015. The Global Monitoring Report on Financial Protection in Health 2019 reported identical estimates to ours-−1.1 and 0.1% at the 10 and 25% thresholds of total household consumption. However, it must be added that the report used econometric modeling techniques to analyze input data for 2012, hence the similarity (1). Other studies that have assessed the effects of health insurance on catastrophic payments reported incidences between 1.3 and 36% (17, 26–30, 36). The differences in results to ours are expected, considering that these studies were limited to comparing incidences between insured and uninsured groups and for certain services among relatively smaller sample populations (17, 26–28). Generally, methodological differences in the measurement of household expenditures or incomes, number of expenditure items covered, and the discretionary use of thresholds often pose challenges for direct comparisons of the results on CHEs between studies (7, 8, 13, 16, 25, 30, 34), as these factors often explain any differences. This, by extension, includes cross-country comparisons as well (7, 13, 24, 25). We acknowledge the FRP implications of our study findings below.

Our results confirm a sustained declining trend in the general population incidence of CHEs in Ghana over time. Evidence suggests that the introduction of the state-subsidized NHIS has played a critical role in that regard. A number of studies that have evaluated the effect of health insurance on OOP health payments by households consistently found that the level of incidence of large unexpected medical payments has decreased among the insured (17, 28)—for instance, Aryeetey et al. (28) found that the NHIS was protective against household OOP expenditures by 86% (28). The scheme has equally been shown to be protective against financial catastrophe with an even much stronger effect among the poorest households (17, 28–30). Relative to the uninsured, Navarrette et al. (30) found that the insured were 7% less likely to incur catastrophic OOP expenditures. Drawing on the evidence from their study, Nguyen et al. (17) observed that, as a social protection mechanism, the scheme is achieving one of its core objectives of extending FRP coverage to a significant section of the population. This assertion is corroborated by evidence from Asia and Latin America where social health insurance schemes similar in design and strategy to the Ghana scheme have provided a level of cover against catastrophic payments to the populations in the lower- and upper-middle-income countries in these regions (3, 4, 8, 10).

A deeper dive, however, revealed some concerning issues: a higher concentration of the incidence of financial catastrophe among the poorest households and significant inequalities in catastrophic payments between the poorest and richest households. Our results therefore reaffirm that, as reported by Zhang et al. (15), general declines masked socio-economic inequalities in the degree to which households experienced financial catastrophe in Ghana. This is implicit of three main issues.

First, wealth-related inequalities in catastrophic payments trap the poorest households in a vicious cycle of financial hardship anytime they come into contact with the health system (17, 25, 28). In SSA, relative to the medical costs, non-medical-related costs associated with access of healthcare services have been documented as larger and contribute more into pushing households into incurring catastrophic payments. The individual and cumulative effects of especially transportation costs, lost incomes, non-routine tests, and poor quality of healthcare heighten the susceptibility of the poorest households to CHEs (13). For these households, the unplanned and unpredictable nature of these expenses may result in trade-offs leading to cuts in the consumption of some other basic necessities such as food and education. This can cause serious disruption to the other social well-being aspects of household life (7, 13, 16, 17). Furthermore, as observed by Njagi et al. (13), wealth-related inequalities in the incidence of CHEs in the poor resource settings of SSA exacerbate inequities in the access of health services for the lower quintiles of the populations. Therefore, to reduce these inequalities in financial catastrophe to insignificant levels in Ghana, increased FRP coverage must simultaneously be pursued along with improved health services coverage (7). It may also require the introduction of supplementary cost-subsidizing schemes to complement the NHIS. These strategies should be pursued within the long-term goal of systematic elimination of OOP payments as share of total health expenditures for the minority poor households specifically and the majority poor in general (7, 8, 37, 38).

Second, the general climate of inadequate public spending on health in Ghana undercut the effectiveness of the FRP policies. The insufficient public funding for healthcare service delivery interacts with a high degree of economic informality (20, 37, 39) to elevate the vulnerability of poor households to catastrophic payments (37, 39), as the greater share of the costs of healthcare services is placed on households (11, 12, 39). The inverse relationship between public spending on health and CHEs where low public sector investments in health result in increased incidence of financial catastrophe has been established (1, 7, 8). In this regard, the low and declining trend in government allocations to health in Ghana (15, 40) has adversely impacted the extension of FRP coverage to the poorest households (40). The effort by the health system to raise more funds has relegated adequate FRP coverage for the poor to a peripheral issue (40). Therefore, it is imperative that, as share of the gross domestic product, the government increases its allocations to health. The NHIS, for instance, can serve as the vehicle for increased public spending on health (1). Channeling public spending through the existing community-based mutual health organizations or targeting them with financial incentives can also serve as an additional safety net layer against catastrophic payments for the poor segments of the population (13). This is a critical step if the government wants to shape a pro-poor pattern of public health spending in the country and of interest to LMICs in general (1).

Third, low enrolment of the poor onto the NHIS deprives them of its protective effects even if these benefits are limited. Enrolment onto the Ghana NHIS has been shown to be pro-rich, contrary to its establishing objective. This has been attributed to the poor rationalization and weak implementation of the exemptions policy, leading to poor coverage for the bottom quintiles (40, 41). Although prepayment mechanisms such as the NHIS do not provide full immunity against catastrophic payments (3, 8, 17, 21), the low coverage nonetheless deprives poor households of the scheme's protective effects against especially large OOP medical payments. Furthermore, the unofficial co-payment for some services covered by the scheme's benefit package, such as consultation fees and medicines, impact the poor the most. This contributes to disproportionately intensifying the incidence of financial catastrophe among these households (17, 36, 42). The interaction of low coverage and unofficial co-payments weakens the FRP sensitivity of the scheme for poor households (1, 3, 4, 10, 11). Granted that insurance enrolment is a poor indicator of FRP coverage (8), it is still important that particular attention be paid to Ghana's coverage policy under the scheme to ensure that it is equity sensitive to the poor (40, 41).

The findings of this study should be discussed in respect of the following limitations: Analysis of financial catastrophe using survey data is prone to errors of underestimation due to both underreporting and zero reporting by the poor on their health expenditures and the coping mechanisms adopted to deal with hardships resulting from health payments (7, 8, 16, 32). Also missing in survey-based datasets is information on economic catastrophe as a consequence of lost earnings due to illness shocks (23). Underestimation therefore weakens the predictive power of OOP payments and CHEs (12, 16, 37). The survey data is also prone to recall bias where the established recall period can impact the information provided on the frequency and magnitude of health payments (8, 13, 32, 43–45). Furthermore, survey data, compared with production level data, tend to miss payments made to informal providers, including other associated indirect costs in the course of healthcare seeking (8, 11, 13). These limitations notwithstanding, we estimated the incidence and intensity of financial catastrophe due to OOP health payments for both total household consumption and non-food consumption using a range of thresholds in accordance with the general guiding principles in empirical literature (11, 12, 16, 25, 43–45). The results also reveal, even if partially, the general situation of CHEs and FRP in Ghana (16). The use of a nationally representative dataset for our analysis directly leads to national estimates of the incidence, intensity, and distribution of CHEs (8).

## Conclusion

The results of this study confirmed the declining trend in the general incidence of CHEs in Ghana. However, the incidence remained disproportionately concentrated among the poorest households, which is instructive of gaps in FRP coverage. While a health insurance has been useful, it does not seem to guarantee adequate FRP coverage for the poor. The poorer households continue to benefit less and are more likely to be at risk of incurring catastrophic payments compared with the rich. Efforts at FRP must go beyond a simple scheme of introducing health insurance. It is important that attention be directed toward the continued monitoring of catastrophic payments to provide timely and policy-relevant information. This will engender corrective prescriptions to the health-financing policies and system to address the specific issues on financial catastrophe faced by the poorer sections of the population. In the meantime, the Ghana NHIS must strengthen its targeting and enrolment of the poorest households to reduce their vulnerability to catastrophic payments.

## Data Availability Statement

Publicly available datasets were analyzed in this study. This data can be found here: http://www.statsghana.gov.gh/nada/index.php or https://open.africa/dataset/ghana-living-standards-survey-glss-7-2017.

## Author Contributions

FS, AS, and KT-A conceived the study. FS and AS drafted the manuscript. KT-A prepared the data. KT-A and FS analyzed the data. All authors read and approved the final manuscript.

## Conflict of Interest

The authors declare that the research was conducted in the absence of any commercial or financial relationships that could be construed as a potential conflict of interest.

## Publisher's Note

All claims expressed in this article are solely those of the authors and do not necessarily represent those of their affiliated organizations, or those of the publisher, the editors and the reviewers. Any product that may be evaluated in this article, or claim that may be made by its manufacturer, is not guaranteed or endorsed by the publisher.

## Abbreviations

ADePT: Automated DEC Poverty Tables
GLSS: Ghana Living Standards Survey
NHIS: National Health Insurance Scheme.
