# Supplementary material for: An Analysis of Catastrophic Out-of-Pocket Health Expenditures in Ghana
Source: Front Health Serv. 2022 Mar 22;2:706216. doi: 10.3389/frhs.2022.706216 (PMC10012771; doi:10.3389/frhs.2022.706216)
Supplement: Supplementary file 1 [file Data_Sheet_1.ZIP › Figures/Figure 2.docx]

**Figure 2: Health payment shares by quintiles**

***Source*: Authors.**
